# Pediatric Multiple Sclerosis: Current Concepts and Consensus Definitions

**DOI:** 10.1155/2013/673947

**Published:** 2013-11-02

**Authors:** Joaquin A. Pena, Timothy E. Lotze

**Affiliations:** Baylor College of Medicine, Houston, TX 77030, USA

## Abstract

Multiple sclerosis (MS), a chronic inflammatory autoimmune disease of the central nervous system (CNS) commonly diagnosed in adults, is being recognized increasingly in children. An estimated 1.7%–5.6% of all patients with MS have clinical symptoms before reaching the age of 18 years. In comparison with adults, the diagnosis of MS in children can be more difficult, being dismissed or misdiagnosed as other clinical disorders. Although adults and children share basic aspects of the disorder, children have distinctive clinical features, neuroimaging, laboratory, and courses of the disease. The 2010 McDonald criteria have simplified the requirements for establishing the diagnosis of MS and have been proposed to be applicable for the diagnosis of pediatric MS, mainly in children 12 years and older. This paper describes the distinctive features of common pediatric demyelinating disorders, including MS, and summarizes the most recent advances based on the available literature.

## 1. Introduction

Multiple sclerosis (MS) is a chronic inflammatory disease of autoimmune nature, characterized by demyelination and axonal loss. MS commonly affects young adults and is considered a rare occurrence in children younger than 18 years of age. However, several studies have indicated that at least 5% of the total population with MS is composed of pediatric patients [1, 2]. Within the pediatric age group, the incidence is highest in those between 13 and 16 years of age. A small, but important, subgroup is younger than 10 years of age [3].

In 2007, an international committee proposed provisional consensus definitions that included a range of clinical and laboratory findings to facilitate unification of criteria for accurate diagnosis and to encourage and promote clinical research in pediatric demyelinating disease [4]. The original definitions have been recently reviewed and updated [5]. These unified criteria have allowed for progress to be made in the advancement of understanding the etiology, clinical manifestations, course, and neuroimaging findings of pediatric MS and other demyelinating disorders of the central nervous system (CNS). However, recognizing distinctive features of different demyelinating disorders to achieve better diagnostic certainty and optimal treatment remain challenging.

## 2. Demographics

MS mainly affects individuals between the ages of 20 and 40 years, with a peak incidence at the age of 30 years. Population studies and case-control series show that between 1.7 and 5.6% of the MS population is younger than 18 years of age [1, 2, 6, 7] and that onset before 10 years of age occurs in less than 1% of all multiple sclerosis cases [2, 7]. The global incidence of pediatric MS is unknown, and the few epidemiological studies exhibit variable results. In a California pediatric cohort, the reported incidence was approximately 0.51 per 100,000 people years [8]. A Canadian surveillance study of initial demyelinating events occurring in subjects younger than 18 years of age, including the first event of MS, neuromyelitis optica (NMO), optic neuritis (ON), acute disseminated encephalomyelitis (ADEM), and transverse myelitis (TM), yielded an incidence of 0.9 per 100,000 people [9]. Another nationwide prospective study in The Netherlands reported an annual incidence of ADS of 0.66/100,000 [10]. Epidemiological studies have determined that the place of residence during childhood is a determinant factor for the development of MS. Adolescent and younger immigrants less than 15 years of age acquire the MS risk that exists in the area to which they move, especially when they move from areas where MS is rare to regions of high prevalence [11].

With regard to gender in pediatric MS, the ratio varies when age is taken into account. In subjects older than 10 years of age and adolescents, females predominate from 2.1 : 1 to 3 : 1, respectively. However, for those younger than 10 years of age, the female-to-male ratio ranges from 0.8 : 1 in children younger than 6 years of age to 1.6 : 1 in patients between 6 and 10 years of age [12].

Unlike the adult population, in whom MS usually affects non-Hispanic whites, pediatric MS shows greater racial and ethnic variability in North America. Chitnis et al. [13] reported not only a greater percentage of African American pediatric patients at a clinic in Boston compared with adults (7.4% versus 4.3%, resp.), but also a more severe clinical presentation for this ethnic group. At a center in Canada, most of the pediatric patients with MS had diverse ethnic backgrounds, including Caribbean, Asian, or Central and Eastern European [11]. The reasons for this ethnic and racial diversity have not been fully elucidated; however, various influences of genetic and environmental, as well as migration, with changing regional demographics factors, may play a role in North America [8, 14, 15]. Whether environmental risk factors for MS are becoming more prevalent during childhood among certain ethnicities or a shift is reflected in the ethnic distribution of general populations from which these cohorts were obtained remains unknown. The population-based cohort study of Southern California children showed a higher incidence of MS in black compared with white and Hispanic children, suggesting that the prevalence of environmental or genetic risk factors may be more common in black children [8].

Other potential environmental factors that contribute to the occurrence of MS include inadequate exposure to sunlight, vitamin D deficiency, viral infections, and exposure to cigarette smoke [16–31].

Usually, MS occurs more commonly in temperate regions, where exposure to ultraviolet light is limited [16]. Ultraviolet radiation is known to induce the synthesis of intraepithelial vitamin D. Currently, vitamin D is considered to be a powerful hormone involved in multiple biological processes, including self-immune recognition. 1,25-Dihydroxyvitamin D_3_, the active form of vitamin D, is a potent immunomodulator that plays key roles in innate and acquired immunities [17]. It downregulates dendritic cells and prevents the proliferation and enhances apoptosis of activated B cells [18, 19]. Lower levels of vitamin D have additionally been associated with increased risk of relapse among patients with relapsing-remitting MS (RRMS) or clinically isolated syndrome (CIS) [19]. In one recent study of pediatric MS, researchers found a 34% decrease in attacks for every 10 ng/mL increase in the level of circulating vitamin D [20]. Similarly, another study showed that each 10 ng/mL higher level of 25-hydroxy vitamin D was associated with a 15% lower risk of acquiring a new T2 lesion and a 32% lower risk of acquiring a gadolinium-enhancing lesion [21].

The pediatric population presents a unique opportunity to study the role of viruses in the development of MS, given the lower total number of pathogen exposures in a young host relative to adults. In addition, the serial novel exposure of children to common viral antigens and close temporal relationship between infection and the onset of pediatric MS provide opportunities to discover the relationship between disease and pathogen [14]. The shorter time lag between putative exposures and disease onset in pediatric MS patients may provide insight into specific environmental factors and/or a particular genetic susceptibility in the pediatric MS population. Viral infections, particularly remote infections with the Epstein-Barr virus (EBV), have been consistently associated with MS in adults and recently documented in more than 85% of children with MS [22, 23]. Banwell et al. [24] compared 137 children with definite MS and controls of the same age and found no differences between the two groups with respect to seropositivity to cytomegalovirus (CMV), herpes simplex type 1 virus, varicella zoster (VZ), and parvovirus B19. In contrast, EBV seropositivity was associated with an increased risk of developing MS in childhood. Another study with 147 children suffering from MS also showed EBV seropositivity more prevalent in patients than in controls (99% versus 72%, *P* = 0.001) [25]. Numerous observations have supported the possibility of multifaceted gene-environment interactions, although only a few have been reported for MS, and those are unconfirmed. The strongest genetic risk factor for MS, HLA-DRB1, is a coreceptor for EBV entry into B cells. In a recent retrospective study, EBNA-1 was associated with increased odds for developing MS in analyses adjusted for age, sex, race, ethnicity, and HLA-DRB1*1501/1503; a remote infection with CMV was associated with a lower risk of developing MS [26]. These findings suggest that a complex interplay may exist between various viral infections acquired during childhood and the risk of developing MS. The combined results of these studies do not yet establish if EBV and/or other infections predispose one to contract MS or if a shared immunogenetic susceptibility toward a symptomatic infection and MS may exist. Moreover, common environmental factors also may trigger both infectious mononucleosis and MS [27]. Further studies are needed to better identify risk factors for MS susceptibility and their interactions, which might lead to development of individualized preventive strategies and new treatments.

The role of some immunizations, especially hepatitis B vaccine, and the subsequent development of MS also have been investigated. Mikaeloff et al. [28] in a French study found no evidence of increased risk of developing a first episode of MS up to 3 years after receiving vaccination. In a second study by the same authors, no evidence was found of any increased rate of relapse after a first demyelinating event when patients were subsequently vaccinated against hepatitis B or tetanus [29]. In a carefully performed case-control analysis, these investigators [30] showed a trend for the Engerix B vaccine to increase the risk of MS in the long term. This did not reach statistical significance, and these results require confirmation.

The same research group assessed the likelihood of developing MS after passive exposure to cigarette smoke in French children. They compared 129 children with MS with 1,038 controls by age, sex, and place of residence. The authors found that the risk of having a first episode of MS in individuals exposed to smoking habits of parents was more than twice that observed in individuals whose parents were nonsmokers, and this risk was even greater in those with prolonged exposure of 10 or more years [31].

## 3. Etiology

As with certain autoimmune diseases, the trigger mechanism of MS in childhood is unknown. The etiology of MS is thought to reflect a complex interplay between host genetic factors and environmental exposures. Still to be determined is how the various factors involved lead to the resulting demyelination and axonal loss that correlate with progression of the disease and neurologic disability. At this point, the literature offers some leading theories that attempt to explain the pathophysiological changes that cause MS. For instance, the largest genome-wide genetic association screens have revealed multiple disease-associated genes that are involved in the immune system function [32, 33]. The major histocompatibility complex exerts the greatest influence on the risk of developing MS followed by other immune genes. Traditionally, T cells were considered the main factor responsible for the attack against CNS elements, particularly myelin. The most recent evidence has revealed a more complex picture in which B cells, antibodies, and the innate immunity also participate in the tissue damage that involves not only myelin but also axons, cortical neurons, and nodes of Ranvier [34]. Despite the sufficient body of evidence on the pathology and neurobiology of MS, the precise characterization of the mechanisms involved in the pathogenesis of MS raises more questions than answers. Autoimmune targets of this widespread injury remain unknown, and one of the current unsolved questions is whether the primary autoimmune attack is the initial trigger (“outside-in model”) or if the MS process begins with a cytodegeneration focused on the oligodendrocyte-myelin complex that results in a reactive inflammatory CNS disorder (“inside-out model”) [35]. The current body of scientific information is consistent with either model, but the need is to understand how these key components work, taking into account the implications for therapeutic design.

Compared to the adult population, few studies in pediatric MS have examined markers of axonal damage in the cerebrospinal fluid (CSF). However, Rostasy et al. [36] presented a group of pediatric patients with MS clinical symptoms displaying elevated levels of Tau protein in the CSF, indicating increased damage to the CNS. The discovery of autoantigens that are expressed by both glial and neuronal cells indicates that an immune attack originally directed against the glial component also can target the neuronal component and vice versa in early events in the human disease [36]. Recent reports have identified autoantibodies to the axoglial membrane proteins neurofascin and contactin in patients with established RRMS [37–39]. More recently, in a study of CSF samples collected from children during initial presentation of acute demyelinating syndromes, levels of nodal/paranodal assembling proteins were significantly higher in the children who ultimately developed MS compared to the monophasic group [40]. These findings complement the view that, as in adults, axoglial apparatus molecules have utility as biomarkers of MS injury and are implicated in early disease mechanisms [40]. A dysfunction of the axoglial interactions possibly leads to loss of trophic support for oligodendrocytes, which in turn may express stress proteins that incite a targeted immune response [40, 41]. Intensive efforts are needed in the field of biomarkers to improve the diagnosis, determine prognostic factors, and identify markers to monitor the clinical course and response to disease-modifying therapies [42]. The ability to perform in-depth analyses of genomes, transcriptomes, proteomes, and metabolomes remains a promising avenue for discoveries of biomarkers in MS.

## 4. Diagnosis

### 4.1. First Demyelinating Event (Clinically Isolated Syndrome (CIS))

The diagnosis of MS in children is a process that begins with a first event of acute demyelination. Hence, it is highly advisable to determine whether the patient will develop subsequent events compatible with MS or if the event is a self-limited disorder. The first attack of demyelination, termed *clinically isolated syndrome* (CIS) or *acquired demyelinating syndrome*, is characterized by a clinical monofocal or polyfocal episode of presumed inflammatory demyelinating cause with acute or subacute onset in the absence of encephalopathy that cannot be explained by fever or systemic illness and that does not meet the 2010 MS McDonald criteria on a baseline MRI [5, 43] (as shown in List 1). CIS can be characterized as clinically monofocal, affecting a localized part of the CNS (ON, brainstem syndrome; TM, hemispheric syndrome), or clinically polyfocal (localizing to multiple sites in the CNS). In a published series of 117 children with acute demyelination and initial monofocal symptoms, 43% were diagnosed with MS, compared to 21% of children with polyfocal features after a follow-up period of 54 months [44]. The likelihood of developing MS following a first event is extremely low in children with an otherwise normal brain MRI [5, 45, 46]. 

*List 1: Clinical Criteria for Pediatric MS and CNS Demyelinating Disorders* [5]

*Pediatric Clinically Isolated Syndrome (CIS)*

- A monofocal or polyfocal clinical neurological event with presumed inflammatory demyelinating cause.
- Absence of encephalopathy that cannot be explained by fever.
- Absence of previous clinical history of CNS demyelinating disease.
- Other etiologies have been excluded.
- The most recent 2010 revised MS McDonald criteria on a baseline MRI are not met.

*Monophasic ADEM*

- A first polyfocal clinical neurological event with presumed inflammatory cause.
- Encephalopathy that cannot be explained by fever is present.
- No new symptoms, signs, or MRI findings after three months of the incident ADEM.

*Multiphasic ADEM*

- A new event of ADEM three months or more after the initial event.
- Can be associated with new or reemergence of prior clinical and MRI findings.
- Timing in relation to steroids is no longer relevant.

*Pediatric Multiple Sclerosis*

- Two or more clinical events separated by more than 30 days and involving more than one area of the CNS.
- A single clinical event plus a baseline MRI evidence for DIS and DIT that meets the recent 2010 revised McDonald criteria.
- ADEM followed more than three months later by a nonencephalopathic clinical event with new lesions on brain MRI consistent with MS.

*NMO*

 All required
  - optic neuritis,
  - acute Myelitis.
 At least two of these three criteria are considered:
  - MRI evidence of a contiguous spinal cord lesion (3 or more segments in length),
  - brain MRI nondiagnostic for MS,
  - antiaquaporin-4 IgG seropositive status.

### 4.2. Optic Neuritis

Although ON in children may appear as a clinically isolated syndrome, other cases of ON are associated with ADEM, MS, NMO, and various other disorders, including inflammatory and infectious conditions. Alternatively, certain genetic conditions, vascular malformations, and compressive orbital tumors can mimic the features of an inflammatory optic neuropathy, necessitating careful investigation. Accordingly, the initial workup should be extensive, including neuroimaging and serologic studies to facilitate the differentiation. Imaging of the brain and orbits with MRI using specific sequences including T2-weighted orbital fat suppression can support the diagnosis of ON with hyperintensity and enlargement of the affected optic nerve. Optic nerve enhancement on T1-weighted sequences following administration of gadolinium is also consistent with an acute inflammatory event.

ON can be unilateral or bilateral. In one study, unilateral ON was observed in 58% of children, compared with a bilateral involvement in 42% of cases [45]. Although initial visual loss was severe in nearly 70% of this group of pediatric patients, 83% of them attained an excellent visual recovery (better than 20/40). As previously noted, ON may occur in isolation as a monofocal clinically isolated syndrome, or it may be associated with other polyfocal acquired demyelinating disorders.

The risk of developing MS after having an isolated episode of ON in childhood has been reported to range between 10% and 56% [45, 47]. Many factors, including the absence of unified definitions, access to neuroimaging, small number of patients, and duration of followup, may explain these widely differing figures. Retrospective case series have examined the prognostic use of magnetic resonance imaging (MRI) in the development of MS following ON. For instance, in the Wilejto et al. study [45] of 36 children with ON, the presence of one or more white matter lesions extrinsic to the optic nerves was associated with a 68% risk for developing MS during the next 2.4 years. More recently, Alper and Wang [48] reported that 23% of pediatric patients with ON eventually developed MS within 6 years in their study and found a strong correlation between a normal MRI and a monophasic clinical presentation. For example, MS was diagnosed in 42% of children with an abnormal MRI, whereas 93% of children with normal MRIs remained relapse-free. Consequently, the presence of ON and associated MRI abnormalities increases the likelihood of developing MS.

### 4.3. Acute Transverse Myelitis

TM may manifest as a monofocal clinically isolated syndrome or be associated with ON, ADEM, or as a component of polyfocal clinically isolated syndrome. TM can be either segmental with involvement of individual vertebral segments of the spinal cord or longitudinally extensive, which is defined as acute transverse myelitis involving 3 or more continuous spinal cord segments in length. The outcome in children with TM is variable. In several series, a complete recovery was reported in 33% to 50% of patients and poor prognosis in approximately 10% to 20% of cases [49, 50].

The risk of MS developing in patients with isolated TM is low. Only one of 47 children with TM followed for a period of 8 years had MS [51]. In the Canadian prospective study, 21% of the children with acquired demyelinating syndrome presented with acute TM, which represented the first clinical event in approximately 10% of children with MS [9]. Although acute TM is a rare presenting symptom in pediatric MS, those children displaying patchy hyperintense T2 signals between 1 and 3 spinal segments or oligoclonal bands in the CSF have the greatest risk for developing MS within this group [49–51].

### 4.4. Acute Disseminated Encephalomyelitis

ADEM, defined as polyfocal neurological deficits of presumed inflammatory and demyelinating cause in association with encephalopathy, is usually a monophasic event [4]. This disorder affects mainly children younger than 10 years of age and usually occurs after they have had viral infections or rarely in association with recent vaccination. A comprehensive workup, including studies of infectious and neurometabolic causes, neuroimaging of the brain and spinal cord, analysis of the CSF, and neuroimmune tests, may help to differentiate ADEM from other disorders [47, 52]. After an ADEM event occurs, the clinical manifestations and neuroimaging findings can fluctuate during the next 3 months and are considered to be part of the same event, rather than separate events. The occurrence of a second event characterized by clinical encephalopathy plus polyfocal neurological deficits at least 3 months after the first episode irrespective of steroid treatment is characterized as multiphasic disseminated encephalomyelitis (MDEM) [5]. Relapsing disease that follows ADEM beyond a second encephalopathic event currently suggests a chronic disorder that often predates the diagnosis of MS or NMO [53, 54]. Some studies have suggested that 18% to 29% of patients with ADEM as their first demyelinating attack progress to MS [47, 55]. However, in a recent prospective study following the definitions proposed by the International Pediatric Multiple Sclerosis Study Group (IPMSSG) on children with ADEM, only 6% developed MS in a 9-year followup [53].

Typical MRI characteristics of ADEM are large, usually at least 2 cm, hyperintense asymmetric lesions, disseminated and confluent, involving white matter, cortex, and the deep grey nuclei with gadolinium enhancement. Recently Callen et al. [56] proposed several MRI findings to better differentiate ADEM from MS. Most patients with ADEM show (a) a diffuse bilateral pattern, (b) absence of black holes, and (c) fewer than two periventricular lesions (sensitivity 81%, specificity 95%). As a consequence, the diagnosis of ADEM is based only on the combination of clinical and neuroimaging findings and exclusion of disorders that resemble this entity.

In children younger than 12 years with features of ADEM to include encephalopathy and polyfocal neurological deficits, application of the revised 2010 MS McDonald criteria for dissemination in space and time on initial MRI is considered inappropriate, and continued follow-up of clinical and MRI findings is needed to confirm a diagnosis of MS [5].

### 4.5. Neuromyelitis Optica (NMO)

Neuromyelitis optica (NMO) is an uncommon inflammatory demyelinating disorder characterized by severe acute transverse myelitis (TM) with simultaneous or sequential unilateral or bilateral optic neuritis (ON). Usually reported in adults and rarely in children, NMO has been considered an exceptional manifestation of multiple sclerosis (MS). However, the discovery of a highly specific aquaporin-4 (AQP4) autoantibody (AQP4-IgG) has demonstrated that NMO is a distinct pathophysiological disorder [57].

Over the last five years a better understanding of pediatric NMO has emerged. A median age of onset of 10–14 years and strong female predominance have been observed [54, 57–59]. Pediatric NMO spectrum can either be monophasic or manifest clinical relapses of ON or TM. Relapsing attacks of ON and TM separated in time are more common, and up to 80% of this group of patients is AQP4-IgG seropositive. Relapsing NMO tends to progress more slowly in children than in adults [60], and clinical relapses of NMO can resemble features of ADEM to include the presence of encephalopathy and large hemispheric lesions on MRI [54, 61].

Diagnostic workup for NMO includes brain and spinal cord MRI and serum AQP4-IgG testing which is 99% specific and 60%–70% sensitive even in pediatric patients [62]. Forty-two percent of children with features of NMO may display serologic (76%) or clinical evidence of systemic lupus erythematosus, Sjogren syndrome, or other autoimmune diseases [58].

Standard CSF analysis during an NMO attack may show pleocytosis with significant number of neutrophils and eosinophils and/or elevation of proteins; oligoclonal bands are generally absent [57, 60, 61].

Current diagnostic criteria are summarized in List 1. Features that suggest NMO or an NMO-spectrum disorder include (1) presence of longitudinally T2-hyperintense spinal cord lesions extending for greater than 3 vertebral segments, (2) optic neuritis, which may have a greater risk of residual deficit compared to ON associated with MS, and (3) brainstem symptoms to include intractable nausea/vomiting, vertigo, hearing loss, facial weakness, trigeminal neuralgia, diplopia, ptosis, and nystagmus [5].

### 4.6. Pediatric Multiple Sclerosis

According to international consensus clinical criteria, pediatric MS is defined by multiple episodes of demyelination of the CNS separated by time and space as specified in adults, eliminating any lower age limit [4, 5]. Therefore, pediatric MS can be diagnosed in patients younger than 18 years with two episodes of CNS demyelination separated by more than 30 days and involving more than one area of the CNS. The consensus is that clinical and radiological criteria of dissemination in time and space must be met [5, 43]. In children aged 12 years and older presenting with an acute event, some typical findings on a baseline MRI may facilitate establishing an early diagnosis when the observed changes are consistent with dissemination in space and time [5, 43].

A high sensitivity (84%) and specificity (93%) of T1 hypointense lesions and T2 periventricular lesions have been recently confirmed and validated as strong early predictors of MS diagnosis in children with acquired demyelinating syndrome (ADS) [63]. As noted earlier, most children with a single demyelinating attack of the CNS will not have recurrences, and only the assessments of clinical investigations, such as neuroimaging, analyses of the CSF, and other laboratory tests, can provide more accurate information regarding which children are at higher risk for developing MS among those who have a single monophasic event. The objective demonstration of dissemination of lesions in both space and time, based on either clinical findings alone or a combination of clinical and MRI findings, remains the core requirement for establishing the diagnosis of MS (List 1).

Most patients with pediatric MS present with a relapsing-remitting course and have much higher relapse rates compared to adults. Gorman et al. [64] have reported that the annualized relapse rate in the pediatric group was significantly higher than that in the adult-onset group (1.13 versus 0.40; *P* < 0.001). This higher rate of early relapses in pediatric MS may be related to different immune activation or levels of cells and cytokines in the CNS. However, the result may have been influenced by referral, since large tertiary referral centers may see patients with a more aggressive disease course. Further prospective studies of early relapse rate in children from first attack are required. Adolescents generally have a second clinical attack within 12 months after the first event, whereas the younger children have a greater time interval between the first and second attacks [7]. Most pediatric patients have complete recovery after their first attacks. A higher risk of experiencing permanent disability seems to be linked to the increased relapse rate within the first 2 years of the disease in pediatric patients [1, 7]. In general, disease progression is slower in pediatric MS, recovery after a clinical exacerbation is shorter in children compared to adults, and a lower proportion of children are classified with progressive forms of the disease [1, 7].

### 4.7. Cognitive Impairment

Available data suggest that approximately one-third of children and adolescents with MS experience cognitive impairment, defined as having at least one-third of completed test scores falling 1 standard deviation or more below published normative data. Areas of cognitive deficit can vary but often include attention and speeded processing, visuomotor functions, memory, and language [47, 65, 66]. Receptive language and verbal fluency are often more affected in pediatric compared with adult MS patients in whom the aspects of language are usually preserved. Interestingly, cognitive impairment was identified in 65 (35%) of 187 children with multiple sclerosis and 8 of 44 (18%) with clinically isolated syndrome in the largest sample studied to date [65]. The most frequent areas involved were fine motor coordination (54%), visuomotor integration (50%) and speeded information processing (35%). This relatively increased proportion of impairment in pediatric MS patients compared to CIS is consistent with the observation that cognitive impairments in children with multiple sclerosis progress over time [67]. Furthermore, the striking difference of cognitive impairment in the early disease course between children and adults with MS may be due to the effects of the inflammatory demyelinating process on the ongoing myelination in the developing brain and neuronal networks [47, 65].

Cognitive dysfunction is a major feature of pediatric multiple sclerosis that can occur at the earliest stages of the disease, interfering with the child's present and future academic performance. In addition, fatigue, depression, and reduced quality of life are important issues in pediatric demyelinating disorders and may occur at a rate up to three times that of controls [66, 67]. Depression or anxiety is present in 50% of children and adolescents with multiple sclerosis, thus interfering with their quality of daily life [65, 66]. Periodic neuropsychological and psychiatric assessment along with the development of interventions for cognitive decline, fatigue, and depression is warranted as part of routine care [68].

## 5. Differential Diagnosis

The diagnosis of pediatric MS is a clinical one, requiring the presence of recurrent episodes of CNS demyelination with supportive ancillary paraclinical data in the absence of another plausible diagnosis. Neuroimaging and CSF analysis features help to establish the diagnosis of pediatric MS. Accordingly, before giving a patient a diagnosis of MS, clinicians should rule out other disorders that may display similar symptoms to include vascular, inflammatory, infectious, metabolic, and neurodegenerative disorders. In a prospective cohort of 332 children meeting clinical criteria for ADS, 20 (6%) were ultimately diagnosed with nondemyelinating disorders [69]. Clinical and paraclinical findings that suggest an alternative diagnosis to initial presentation of MS include fever, encephalopathy, progressive clinical course, involvement of the peripheral nervous system or other organ systems, increased leukocyte count or ESR, markedly elevated pleocytosis or proteinorraquia, and the absence of CSF oligoclonal bands [70]. The combination of peripheral neuropathy and CNS demyelination argue against MS and favor other entities such as leukodystrophies or mitochondrial diseases.

This group of disorders usually exhibits progressive neurologic deterioration in absence of a clear relapsing-remitting disease.

CNS vasculitis is a challenging differential diagnosis of ADS with occasional overlapping features to include optic neuritis, transverse myelitis, and polyfocal supratentorial and infratentorial neurologic deficits [69, 71]. Persistent headache, rarely observed in MS or children with CIS, was present in 4 of the 5 patients with childhood primary angiitis of the CNS in the O'Mahony et al.'s study [69]. Seizures were observed in 4 of the 5 children with childhood primary angiitis compared to only 3 of more than 301 children with CNS demyelination. Focal seizures in the absence of persistent neurological deficits may be associated with CNS malignancy.

The evolution of disease by neuroimaging can help to confirm or exclude an MS diagnosis. White matter abnormalities on MRI in pediatric patients have a wide range of differential diagnoses (List 2). These entities should more often be considered in the younger child or when the presentation is atypical [70]. 

*List 2: Diagnostic Categories to Exclude in Pediatric Multiple Sclerosis*

*Vascular/Inflammatory Disease*

- CNS vasculitis/childhood primary CNS angiitis,
- Stroke,
- CADASIL,
- Autoimmune disease: systemic lupus erythematous, antiphospholipid antibody syndrome, neurosarcoidosis, Sjogren's syndrome,
- Migraine.

*Metabolic/Nutritional*

- Mitochondrial encephalopathy,
- Leukodystrophies,
- B12 or folate deficiency.

*CNS Infection*

- Neuroborreliosis,
- Herpes simplex encephalitis,
- Influenza ANE,
- Viral encephalitis.

*Malignancy*

- Lymphoma,
- Astrocytoma.

Regarding neuroimaging, persistent lesion enhancement and edema or increasing size of lesions over time are considered “red flags” for an underlying malignant condition. Leptomeningeal enhancement uncommon in MS may point to either childhood primary angiitis of the CNS or an inflammatory disorder with meningeal infiltration as neurosarcoidosis [69]). Alternatively, clinical and imaging involvement of basal ganglia is not common in MS and may suggest other diagnosis such as mitochondrial disease, infectious encephalitis, or histiocytosis [69, 70]. A correct and timely diagnosis is vital to lead the children and their families to the appropriate treatment and reduce the potential long-term disability [70].

## 6. Diagnostic Tools

In patients with demyelinating events, evaluation by neuroimaging, analysis of the CSF, visual testing including the visual evoked potentials (VEP), and ocular coherence tomography (OCT) are important diagnostic tools.

### 6.1. Neuroimaging (Brain MRI)

**Figure 1:**
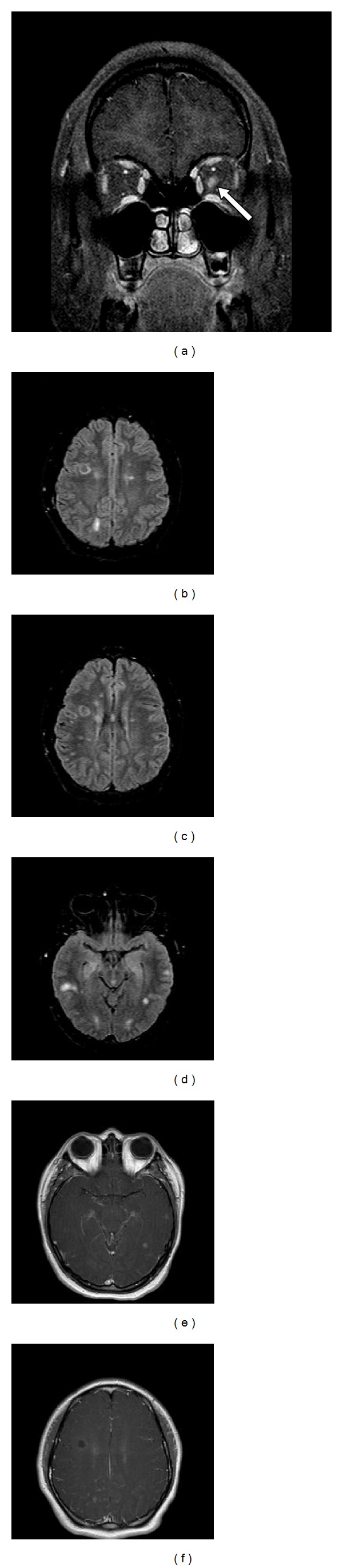
(a) Coronal T1 gadolinium enhanced sequence demonstrating left optic neuritis with enhancement and enlargement of the left optic nerve (arrow). (b) and (c) Axial FLAIR demonstrating typical well-circumscribed ovoid lesions in the juxtacortical and periventricular regions consistent with 2010 McDonald criteria for dissemination in space. (d) and (e) Axial FLAIR and gadolinium enhanced sequences with corresponding asymptomatic enhancing and nonenhancing lesions consistent with 2010 McDonald criteria for dissemination in time. (f) Axial T1 sequence with hypointense lesion associated with acute demyelination and axonal injury.

Currently, MRI is the most important diagnostic tool for evaluating MS in both children and adults, as it has invaluable utility in the recognition of other disorders that may resemble ADEM or MS. MRI findings in MS consist of plaques of demyelination particularly visible on T2-weighted sequences and typically located in the deep white matter, corpus callosum, periventricular zone, and brainstem. T1 sequences may demonstrate “black holes” or hypointense lesions that represent complete tissue loss resulting from a previous inflammatory event (Figures 1(a)–1(f)). Enhancement of active areas of inflammation and blood-brain barrier compromise can be displayed with T1 gadolinium contrast sequences. Tumefactive T2-bright lesions can be seen in up to 0.3 cases per 100.000 per year. Characteristic features that can help to distinguish demyelination from a malignant process include preferential enhancement of the lesional rim facing the lateral ventricles [72, 73].

Retrospective data suggest that children at MS onset have a higher number of total hyperintense T2 lesions in the posterior fossa and overall more gadolinium-enhancing lesions than adults do. In addition, compared to adults, pediatric MS patients tend to have greater resolution of the initial T2 lesion burden on follow-up MRI, suggesting better recovery of demyelination in children [74].

Current diagnostic criteria for MS admit MRI evidence of new lesions over time to substitute for clinical relapses. The most recent revision of the McDonald criteria specifically outlines the applicability for the use of the revised criteria in children and permits the diagnosis of pediatric MS at a first clinical event [43]. According to these criteria, dissemination in space (DIS) can be fulfilled with one or more lesions in at least two of four CNS areas (periventricular, juxtacortical, infratentorial, or spinal cord). Additionally, DIT can also be fulfilled in patients with typical acute demyelinating syndrome with a single MRI study that demonstrates simultaneous presence of asymptomatic gadolinium-enhancing and nonenhancing lesions [5, 43].

In the prospective cohort study by Sadaka et al. [75], the 2010 revised McDonald criteria demonstrated high sensitivity (100%), specificity (86%), positive predictive value (76%), and negative predictive value (100%) for children older than 12 years with non-ADEM presentations. In younger children, these criteria are of less predictive value and not appropriate for application in the context of ADEM-like presentations.

The emerging emphasis on the MRI features in the diagnosis of MS in younger children can be challenging, given the higher incidence of ADEM in this age group and often equivalent imaging features between ADEM and MS in this population with large confluent, ill-defined lesions early in the disease course. This particular phenotype contributes considerably to misdiagnosis of a significant number of patients [76].

### 6.2. Cerebrospinal Fluid (CSF) Analysis

CSF provides valuable information about the inflammatory process of the CNS. Its analysis, which includes cellular profiles, oligoclonal bands, and IgG Index, has been used to define and differentiate MS from other disorders. The profile of the CSF in pediatric MS may vary depending on the child's age. Compared to adolescents, children younger than 10 years of age tend to show more neutrophils in the CSF, and the CSF cellular profile in children tends to disappear in repeated analyses, on average 19 months after the initial examination [77]. The absence of neutrophils in the CSF at the onset of the disease may be a predictive factor of a second and early neurological episode. These observations suggest that the age of the patient exerts a modifying effect on the CSF cellular profile at the beginning of the disorder, which leads to activation of the innate immune system in the early stages or to an immature immune response [77].

CSF cell count and protein are normal in as many as 60% of pediatric patients with MS; the other patients show a discrete increase in the number of white blood cells or proteins [1, 78]. The percentage of pediatric patients with MS who also have oligoclonal bands has been reported to be up to 92%, providing that the spinal fluid is analyzed using isoelectric focusing assays [79, 80]. In some cases, the oligoclonal bands initially can be negative and detected only later in the course of the disease. It has been reported that positive oligoclonal bands may be found in 29% of patients with ADEM [78]. Mikaeloff et al., [55] in a study with 72 children presenting with a first demyelinating event, found that 94% of children with positive oligoclonal bands went on to develop MS. Moreover, only 40% of patients with definitive diagnosis of MS had oligoclonal bands. These results suggest that oligoclonal bands have low sensitivity but high specificity for the development of MS.

### 6.3. Visual Evaluation

Visual deficit may go unnoticed in children with MS. Although ON may be a presenting symptom, a significant number of patients may have subclinical abnormalities of the visual pathway [81]. In fact, the visual pathways frequently are affected in MS, even in patients without visual disturbances. The visual evoked potential has diagnostic utility in pediatric MS, revealing a second focus of demyelination before a second clinical attack occurs [81]. Ocular coherence tomography (OCT), which permits *in vivo* characterization of the tissue structures with higher resolution by quantifying the thickness of the retinal nerve fiber layer containing nonmyelinated axons as well as the macular volume, has been proposed as a useful tool to evaluate patients with demyelinating disorders [82, 83]. The determination of the total macular volume has been suggested as a marker for neuronal loss in patients with MS. Similarly, a correlation between reduction of retinal nerve fiber layer thickness and both brain atrophy (by MRI) and level of disability (by Kurtzke's EDSS score) also has been reported [84, 85]. In children with MS, this tool provides a sensitive demonstration of optic atrophy and, together with the ophthalmological assessment to include visual evoked potentials, provides objective evidence of a previous inflammatory insult to the optic nerve. A recent study on OCT in children reported a significant retinal atrophy in the pediatric population with demyelinating disorders including optic neuritis, MS, and ADEM. Retinal atrophy was found to be more marked in patients with a previous episode of ON [86].

## 7. Treatment

### 7.1. Acute Treatment

Acute relapses of pediatric MS are usually treated with IV methyl prednisolone 20–30 mg/kg (maximum 1 g daily) for 3−5 days followed by oral taper. Available data in adults do not support the need for a corticosteroid taper after completion of pulse corticosteroid therapy. Pediatric patients with recurrent symptoms after discontinuation of intravenous corticosteroids may raise the possible need for an oral taper [87]. If there is an incomplete response or in case of a severe attack, intravenous immune globulin (IVIG) at 0.4 g/kg/day for 5 days or plasmapheresis should be considered.

### 7.2. Preventive Therapy

To date, there have been no randomized control trials of any DMT in the pediatric population, and the use of these treatments is mainly based on several adult clinical trials and small retrospective, observational studies. First-line therapies include intramuscular interferon (IFN)-b1a (300 mcg once a week), subcutaneous IFN b-1a (22 or 44 mcg 3 times a week), subcutaneous IFN-b1 b (0.25 mg every other day), or glatiramer acetate (20 mg/day) [88, 89].

Gradual titration of the interferon dosing over four to six weeks is common practice in children. In published studies, the majority of patients were escalated to full dose, unadjusted for age or body weight. Disease control is not always achieved immediately. Adherence to medication and time to effective dosing should be evaluated if relapses continue. If disease activity continues after 6–12 months of treatment, a change in therapies may be considered. Although there is no evidence-based guidelines as to when to switch therapies, working definitions of breakthrough disease in need of treatment modification from the IPMSSG suggest the following: (1) minimum time of full dose therapy of 6 months and (2) full medication adherence and one of the following: (a) increase or no reduction in the relapse rate or new T2 or enhancing lesion on MRI as compared to previous treatment or (b) ≥2 confirmed MRI or clinical relapses within a 12-month period [90].

Refractory disease may be considered if there are further relapses or silent progression of disease on MRI. There are several new immunomodulatory agents for refractory pediatric MS. These therapies include monoclonal antibody therapy (e.g., natalizumab, daclizumab), chemotherapeutic agents (e.g., cyclophosphamide, mitoxantrone), and oral medications with novel mechanisms of action (e.g., fingolimod, teriflunomide, and dimethyl fumarate (BG-12)). Among this group, only natalizumab, mitoxantrone, fingolimod, and teriflunomide have been approved by the FDA for use in adults with MS.

### 7.3. Challenges regarding Current and Future MS Therapies

Available data suggest that about 40% of pediatric MS patients discontinue treatment owing to intolerance, toxicity, persisting relapses, or nonadherence, supporting a need for developing new therapies in this population. Only well-designed clinical trials and long-term safety monitoring may allow the pediatric patients to benefit from the advances in MS standard of care.

Recent legislation in the United States and Europe has now mandated pediatric studies for new biological products. In Europe, a pediatric investigation plan (PIP) must be submitted to the European Medicines Agency (EMA). Similarly, the Pediatric Research Equity Act (PREA) in the United States requires pediatric studies for any new active molecule, new dosage form, or new route of administration.

A full or partial waiver is possible if the treated condition does not occur in the pediatric population or if studies are not feasible or appropriate or safe for the age group. Additionally, the Best Pharmaceuticals Act for Children (BPCA) in the United States allows for voluntary pediatric drug assessments via written requests issued by the FDA, with the incentive of eligibility of an additional 6 months of market exclusivity [91].

A meeting report on Clinical Trial Summit from the Steering Committee of the International Pediatric MS Study Group (IPMSSG) has been recently published [91]. The academic leaders established guidelines for outcome measures, including clinical, cognitive, and MRI, to be considered in the pediatric MS clinical drug trials. Despite the growing arsenal of therapies that offers substantial promise for pediatric patients, there are some immediate and long-term health risks, and only well-designed, multicenter trials with long-term followup will properly assess accompanying hazards and safety.

## 8. Conclusions

The diagnosis of pediatric MS needs to be considered in those patients in whom optic nerve, sensory, motor, brainstem, and/or cerebellar disturbance are the presenting symptoms. A comprehensive history aided by clinical, neuroimaging, and laboratory clues can help to assure a prompt diagnosis and the exclusion of other neurological disorders. In younger patients, however, a polyfocal presentation with associated encephalopathy may be difficult to distinguish from ADEM. As in adult-onset MS, the MRI features of pediatric MS involve the presence of multiple lesions, mostly in the white matter and typically observed in the periventricular area of the corpus callosum and spinal cord. Children often show more infratentorial lesions, predominantly in the pons, and can have large and tumefactive lesions with perilesional edema. The most recent revision of the McDonald criteria specifically underscores its applicability in diagnosing MS in children older than 12 years and in facilitating the diagnosis at a first clinical attack, providing the criteria for dissemination in space and time are met.

During the last 10 years, new insights regarding the pathology and immunobiology, clinical features, and neuroimaging have increased the ability to better understand pediatric MS. For example, different studies have identified the potential roles of EBV and low vitamin D in the pathogenesis of MS. However, information about the nature of the immune mechanisms involved in pediatric MS and the interactions of risk factors with genetic susceptibility is limited. On the horizon, identification of biomarkers with the promise to predict disease onset and monitor disease course, severity, and response to treatment has led to a renewed and increased interest and may provide important information for the best management of patients.

## References

[1] Duquette P, Murray TJ, Pleines J (1987). Multiple sclerosis in childhood: clinical profile in 125 patients. *Journal of Pediatrics*.

[2] Ghezzi A, Deplano V, Faroni J (1997). Multiple sclerosis in childhood: clinical features of 149 cases. *Multiple Sclerosis*.

[3] Ruggieri M, Iannetti P, Polizzi A, Pavone L, Grimaldi LM (2004). Multiple sclerosis in children under 10 years of age. *Neurological Sciences*.

[4] Krupp LB, Banwell B, Tenembaum S (2007). International Pediatric MS Study Group, Consensus definitions proposed for pediatric multiple sclerosis and related disorders. *Neurology*.

[5] Krupp LB, Tardieu M, Amato MP (2013). International Pediatric Multiple Sclerosis Study Group criteria for pediatric multiple sclerosis and immune-mediated central nervous system demyelinating disorders: revisions to the 2007 definitions. *Multiple Sclerosis Journal*.

[6] Deryck O, Ketelaer P, Dubois B (2006). Clinical characteristics and long term prognosis in early onset multiple sclerosis. *Journal of Neurology*.

[7] Renoux C, Vukusic S, Mikaeloff Y (2007). Natural history of multiple sclerosis with childhood onset. *New England Journal of Medicine*.

[8] Langer-Gould A, Zhang JL, Chung J, Yeung Y, Waubant E, Yao J (2011). Incidence of acquired CNS demyelinating syndromes in a multiethnic cohort of children. *Neurology*.

[9] Banwell B, Kennedy J, Sadovnick D (2009). Incidence of acquired demyelination of the CNS in Canadian children. *Neurology*.

[10] Ketelslegers IA, Catsman-Berrevoets CE, Neuteboom RF (2012). Incidence of acquired demyelinating syndromes of the CNS in Dutch children: a nationwide study. *Journal of Neurology*.

[11] Kennedy J, O’Connor P, Sadovnick AD, Perara M, Yee I, Banwell B (2006). Age at onset of multiple sclerosis may be influenced by place of residence during childhood rather than ancestry. *Neuroepidemiology*.

[12] Banwell B, Ghezzi A, Bar-Or A, Mikaeloff Y, Tardieu M (2007). Multiple sclerosis in children: clinical diagnosis, therapeutic strategies, and future directions. *Lancet Neurology*.

[13] Chitnis T, Glanz B, Jaffin S, Healy B (2009). Demographics of pediatric-onset multiple sclerosis in an MS center population from the Northeastern United States. *Multiple Sclerosis*.

[14] Yeh EA, Chitnis T, Krupp L (2009). Pediatric multiple sclerosis. *Nature Reviews Neurology*.

[15] Chitnis T, Krupp L, Yeh A (2011). Pediatric Multiple Sclerosis. *Neurologic Clinics*.

[16] Mehta BK (2010). New hypotheses on sunlight and the geographic variability of multiple sclerosis prevalence. *Journal of the Neurological Sciences*.

[17] Mowry EM (2011). Vitamin D: evidence for its role as a prognostic factor in multiple sclerosis. *Journal of the Neurological Sciences*.

[18] Chen S, Sims GP, Xiao XC, Yue YG, Chen S, Lipsky PE (2007). Modulatory effects of 1,25-dihydroxyvitamin D3 on human B cell differentiation. *Journal of Immunology*.

[19] Simpson S, Taylor B, Blizzard L (2010). Higher 25-hydroxyvitamin D is associated with lower relapse risk in multiple sclerosis. *Annals of Neurology*.

[20] Mowry EM, Krupp LB, Milazzo M (2010). Vitamin D status is associated with relapse rate in pediatric-onset multiple sclerosis. *Annals of Neurology*.

[21] Mowry EM, Waubant E, McCulloch CE (2012). Vitamin D status predicts new brain magnetic resonance imaging activity in multiple sclerosis. *Annals of Neurology Journal*.

[22] Ascherio A, Munger KL, Lennette ET (2001). Epstein-Barr virus antibodies and risk of multiple sclerosis: a prospective study. *Journal of the American Medical Association*.

[23] Alotaibi S, Kennedy J, Tellier R, Stephens D, Banwell B (2004). Epstein-Barr virus in pediatric multiple sclerosis. *Journal of the American Medical Association*.

[24] Banwell B, Krupp L, Kennedy J (2007). Clinical features and viral serologies in children with multiple sclerosis: a multinational observational study. *Lancet Neurology*.

[25] Pohl D, Krone B, Rostasy K (2006). High seroprevalence of Epstein-Barr virus in children with multiple sclerosis. *Neurology*.

[26] Waubant E, Mowry EM, Krupp L (2011). Common viruses associated with lower pediatric multiple sclerosis risk. *Neurology*.

[27] Pohl D (2009). Epstein-Barr virus and multiple sclerosis. *Journal of the Neurological Sciences*.

[28] Mikaeloff Y, Caridade G, Rossier M, Suissa S, Tardieu M (2007). Hepatitis B vaccination and the risk of childhood-onset multiple sclerosis. *Archives of Pediatrics and Adolescent Medicine*.

[29] Mikaeloff Y, Caridade G, Assi S, Tardieu M, Suissa S (2007). Hepatitis B vaccine and risk of relapse after a first childhood episode of CNS inflammatory demyelination. *Brain*.

[30] Mikaeloff Y, Caridade G, Suissa S, Tardieu M (2009). Hepatitis B vaccine and the risk of CNS inflammatory demyelination in childhood. *Neurology*.

[31] Mikaeloff Y, Caridade G, Tardieu M, Suissa S (2007). Parental smoking at home and the risk of childhood-onset multiple sclerosis in children. *Brain*.

[32] Dyment DA, Ebers GC, Sadovnick AD (2004). Genetics of multiple sclerosis. *Lancet Neurology*.

[33] de Jager PL, Jia X, Wang J (2009). Meta-analysis of genome scans and replication identify CD6, IRF8 and TNFRSF1A as new multiple sclerosis susceptibility loci. *Nature Genetics*.

[34] Derfuss T, Linington C, Hohlfeld R, Meinl E (2010). Axo-glial antigens as targets in multiple sclerosis: implications for axonal and grey matter injury. *Journal of Molecular Medicine*.

[35] Stys PK, Zamponi GW, van Minnen J, Geurts JJ (2012). Will the real multiple sclerosis please stand up?. *Nature Reviews Neuroscience*.

[36] Rostasy K, Withut E, Pohl D (2005). Tau, phospho-tau, and S-100B in the cerebrospinal fluid of children with multiple sclerosis. *Journal of Child Neurology*.

[37] Derfuss T, Parikh K, Velhin S (2009). Contactin-2/TAG-1-directed autoimmunity is identified in multiple sclerosis patients and mediates gray matter pathology in animals. *Proceedings of the National Academy of Sciences of the United States of America*.

[38] Mathey EK, Derfuss T, Storch MK (2007). Neurofascin as a novel target for autoantibody-mediated axonal injury. *Journal of Experimental Medicine*.

[39] Meinl E, Derfuss T, Krumbholz M, Pröbstel AK, Hohlfeld R (2011). Humoral autoimmunity in multiple sclerosis. *Journal of the Neurological Sciences*.

[40] Dhaunchak AS, Becker C, Schulman H (2012). Implication of perturbed axoglial apparatus in early pediatric multiple sclerosis. *Annals of Neurology*.

[41] Van Noort JM, Bsibsi M, Gerritsen WH (2010). *α*B-crystallin is a target for adaptive immune responses and a trigger of innate responses in preactive multiple sclerosis lesions. *Journal of Neuropathology and Experimental Neurology*.

[42] Bennett JL, Owens GP (2012). Cerebrospinal fluid proteomics: a new window for understanding human demyelinating disorders?. *Annals of Neurology*.

[43] Polman CH, Reingold SC, Banwell B (2011). Diagnostic criteria for multiple sclerosis: 2010 revisions to the McDonald criteria. *Annals of Neurology*.

[44] Neuteboom RF, Boon M, Catsman Berrevoets CE (2008). Prognostic factors after a first attack of inflammatory CNS demyelination in children. *Neurology*.

[45] Wilejto M, Shroff M, Buncic JR, Kennedy J, Goia C, Banwell B (2006). The clinical features, MRI findings, and outcome of optic neuritis in children. *Neurology*.

[46] Verhey LH, Branson HM, Shroff MM (2011). MRI parameters for prediction of multiple sclerosis diagnosis in children with acute CNS demyelination: a prospective national cohort study. *The Lancet Neurology*.

[47] Bigi S, Banwell B (2012). Pediatric multiple sclerosis. *Journal of Child Neurology*.

[48] Alper G, Wang L (2009). Demyelinating optic neuritis in children. *Journal of Child Neurology*.

[49] Miyazawa R, Ikeuchi Y, Tomomasa T, Ushiku H, Ogawa T, Morikawa A (2003). Determinants of prognosis of acute transverse myelitis in children. *Pediatrics International*.

[50] Borchers AT, Gershwin ME (2012). Transverse myelitis. *Autoimmunity Reviews*.

[51] Pidcock FS, Krishnan C, Crawford TO, Salorio CF, Trovato M, Kerr DA (2007). Acute transverse myelitis in childhood: center-based analysis of 47 cases. *Neurology*.

[52] Pohl D, Tenembaum S (2012). Treatment of acute disseminated encephalomyelitis. *Current Treatment Options in Neurology*.

[53] Mar S, Lenox J, Benzinger T, Brown S, Noetzel M (2010). Long-term prognosis of pediatric patients with relapsing acute disseminated encephalomyelitis. *Journal of Child Neurology*.

[54] Lotze TE, Northrop JL, Hutton GJ, Ross B, Schiffman JS, Hunter JV (2008). Spectrum of pediatric neuromyelitis optica. *Pediatrics*.

[55] Mikaeloff Y, Suissa S, Vallée L (2004). First episode of acute CNS inflammatory demyelination in childhood: prognostic factors for multiple sclerosis and disability. *Journal of Pediatrics*.

[56] Callen DJA, Shroff MM, Branson HM (2009). Role of MRI in the differentiation of ADEM from MS in children. *Neurology*.

[57] Wingerchuk DM (2010). Neuromyelitis optica spectrum disorders. *CONTINUUM Lifelong Learning in Neurology*.

[58] McKeon A, Lennon VA, Lotze T (2008). CNS aquaporin-4 autoimmunity in children. *Neurology*.

[59] Peña JA, Ravelo ME, Mora-La Cruz E, Montiel-Nava C (2011). NMO in pediatric patients: brain involvement and clinical expression. *Arquivos de Neuro-Psiquiatria*.

[60] Morrow MJ, Wingerchuk D (2012). Neuromyelitis optica. *Journal of Neuro-Ophthalmology*.

[61] Tillema JM, McKeon A (2012). The spectrum of neuromyelitis optica (NMO) in childhood. *Journal of Child Neurology*.

[62] Banwell B, Tenembaum S, Lennon VA (2008). Neuromyelitis optica-IgG in childhood inflammatory demyelinating CNS disorders. *Neurology*.

[63] Verhey LH, van Pelt-Gravesteijnc ED, Ketelslegersc A (2013). Validation of MRI predictors of multiple sclerosis diagnosis in children with acute CNS demyelination. *Multiple Sclerosis and Related Disorders*.

[64] Gorman MP, Healy BC, Polgar-Turcsanyi M, Chitnis T (2009). Increased relapse rate in pediatric-onset compared with adult-onset multiple sclerosis. *Archives of Neurology*.

[65] Julian L, Serafin D, Charvet L (2013). Cognitive impairment occurs in children and adolescents with multiple sclerosis: results from a United States network. *Journal of Child Neurology*.

[66] MacAllister WS, Boyd JR, Holland NJ, Milazzo MC, Krupp LB (2007). The psychosocial consequences of pediatric multiple sclerosis. *Neurology*.

[67] MacAllister WS, Christodoulou C, Milazzo M, Krupp LB (2007). Longitudinal neuropsychological assessment in pediatric multiple sclerosis. *Developmental Neuropsychology*.

[68] Glanz BI, Healy BC, Hviid LE, Chitnis T, Weiner HL (2012). Cognitive deterioration in patients with early multiple sclerosis: a 5-year study. *Journal of Neurology, Neurosurgery and Psychiatry*.

[69] O’Mahony J, Bar-Or A, Arnold DL, Sadovnick AD, Marrie RA, Banwell B (2013). Masquerades of acquired demyelination in children: experiences of a national demyelinating disease program. *Journal of Child Neurology*.

[70] Hahn JS, Pohl D, Rensel M, Rao S (2007). Differential diagnosis and evaluation in pediatric multiple sclerosis. *Neurology*.

[71] Twilt M, Benseler SM (2013). CNS vasculitis in children. *Multiple Sclerosis and Related Disorders*.

[72] McAdam LC, Blaser SI, Banwell BL (2002). Pediatric tumefactive demyelination: case series and review of the literature. *Pediatric Neurology*.

[73] Riva D, Chiapparini L, Pollo B, Balestrini MR, Massimino M, Milani N (2008). A case of pediatric tumefactive demyelinating lesion misdiagnosed and treated as glioblastoma. *Journal of Child Neurology*.

[74] Waubant E, Chabas D, Okuda DT (2009). Difference in disease burden and activity in pediatric patients on brain magnetic resonance imaging at time of multiple sclerosis onset vs adults. *Archives of Neurology*.

[75] Sadaka Y, Verhey LH, Shroff MM (2012). 2010 McDonald criteria for diagnosing pediatric multiple sclerosis. *Annals of Neurology*.

[76] Chabas D, Castillo-Trivino T, Mowry EM, Strober JB, Glenn OA, Waubant E (2008). Vanishing MS T2-bright lesions before puberty: a distinct MRI phenotype?. *Neurology*.

[77] Chabas D, Ness J, Belman A (2010). Younger children with MS have a distinct CSF inflammatory profile at disease onset. *Neurology*.

[78] Dale RC, de Sousa C, Chong WK, Cox TCS, Harding B, Neville BGR (2000). Acute disseminated encephalomyelitis, multiphasic disseminated encephalomyelitis and multiple sclerosis in children. *Brain*.

[79] Ghezzi A, Pozzilli C, Liguori M (2002). Prospective study of multiple sclerosis with early onset. *Multiple Sclerosis*.

[80] Pohl D, Rostasy K, Reiber H, Hanefeld F (2004). CSF characteristics in early-onset multiple sclerosis. *Neurology*.

[81] Pohl D, Rostasy K, Treiber-Held S, Brockmann K, Gärtner J, Hanefeld F (2006). Pediatric multiple sclerosis: detection of clinically silent lesions by multimodal evoked potentials. *Journal of Pediatrics*.

[82] Costello F, Coupland S, Hodge W (2006). Quantifying axonal loss after optic neuritis with optical coherence tomography. *Annals of Neurology*.

[83] Frohman E, Costello F, Zivadinov R (2006). Optical coherence tomography in multiple sclerosis. *Lancet Neurology*.

[84] Gordon-Lipkin E, Chodkowski B, Reich DS (2007). Retinal nerve fiber layer is associated with brain atrophy in multiple sclerosis. *Neurology*.

[85] Henderson APD, Trip SA, Schlottmann PG (2008). An investigation of the retinal nerve fibre layer in progressive multiple sclerosis using optical coherence tomography. *Brain*.

[86] Yeh EA, Weinstock-Guttman B, Lincoff N (2009). Retinal nerve fiber thickness in inflammatory demyelinating diseases of childhood onset. *Multiple Sclerosis*.

[87] Yeh EA (2012). Management of children with multiple sclerosis. *Pediatric Drugs*.

[88] Ghezzi A, Amato MP, Capobianco M (2005). Disease-modifying drugs in childhood-juvenile multiple sclerosis: results of an Italian co-operative study. *Multiple Sclerosis*.

[89] Yeh EA (2011). Current therapeutic options in pediatric multiple sclerosis. *Current Treatment Options in Neurology*.

[90] Chitnis T, Tenembaum S, Banwell B (2012). Consensus statement: evaluation of new and existing therapeutics for pediatric multiple sclerosis. *Multiple Sclerosis*.

[91] Chitnis T, Tardieu M, Amato MP (2013). International Pediatric MS Study Group Clinical Trials Summit: meeting report. *Neurology*.

